# Kinematic and Kinetic Analyses of the Vertical Jump with and without Header as Performed by Para-Footballers with Cerebral Palsy

**DOI:** 10.3390/sports7090209

**Published:** 2019-09-12

**Authors:** Raúl Reina, José L.L. Elvira, Manuel Valverde, Alba Roldán, Javier Yanci

**Affiliations:** 1 Sport Research Centre, Department of Sport Sciences, Miguel Hernández University, 3202 Elche, Spain; rreina@umh.es (R.R.); jose.lopeze@umh.es (J.L.L.E.); mvalverde@umh.es (M.V.); aroldan@umh.es(A.R.); 2 Faculty of Education and Sport, University of the Basque Country, 01007 Vitoria-Gasteiz, Spain

**Keywords:** cerebral palsy football, paralympics, para-sport, biomechanics

## Abstract

Vertical jump is a relevant variable in the classification of football for individuals with cerebral palsy. In this regard, the literature is limited. There are no studies assessing vertical jumping ability through kinematic methods and in more specific football game situations, such as jumps with a header. The goals of the present study were to assess how the modification of jumping conditions (without and with a header) might affect the kinematic and kinetic parameters of counter movement jumping, and whether the functional profiles of the players constrain their ability to jump vertically, both with and without a header. Thirteen male football players with cerebral palsy (27.7 ± 5.7 years old) and different functional profiles participated in this study. All the players performed ten counter movement jumps with arms swing, five headed a ball and five did not. The kinematic parameters were recorded with a 3D motion analysis system, and the kinetic parameters using a force platform. Significantly smaller angles of the hips (*d*_g_ = 0.75–0.79; *p* < 0.01) and knees (*d*_g_ = 1.04–1.15; *p* < 0.05), as well as greater ankle extension (*d*_g_ = −0.71; *p* < 0.05), were observed during the eccentric phase of the jumps with a header. There were also asymmetries between legs in ankle extension during jumps with a header (*d*_g_ = −1.06; *p* < 0.05), which could be an adjustment element for the precision of the jumps (i.e., header action). It should be mentioned that the jumping pattern could be partially affected by the functional profile of football players with cerebral palsy.

## 1. Introduction

Cerebral palsy (CP) is a persistent movement and posture disorder caused by damage to the central nervous system (CNS) during the early period of brain development [1]. It is characterised by changes in physical functional ability, such as alteration of muscle tone, coordination, and posture, deriving from structural, biochemical, or electrical abnormalities of the CNS, which can manifest themselves as a variety of symptoms depending of the affected CNS area [2,3]. There are different forms of CP depending on motor involvement. Muscle spasticity [4] and weakness [5] are the most frequent manifestations.

In the sport context, especially with regard to classification in paralympic sports, the international standard on eligible impairments of the International Paralympic Committee [6] determines three physical impairments associated with CP: (a) hypertonia, where para-athletes have an increase in muscle tension and a reduced ability of a muscle to stretch, (b) ataxia, where the proficiency of para-athletes is constrained by uncoordinated movements, and (c) athetosis, where para-athletes exhibit continual slow involuntary movements. With regard to the affected body region, the Cerebral Palsy International Sports and Recreation Association, classifies ambulatory people with CP as [7]: (a) moderate spastic diplegia, where the function of lower limbs is limited by bilateral spasticity, (b) moderate ataxic or athetoid profile involving the four limbs and trunk, (c) moderate hemiplegia, where one side of the body (right/left arm and leg) is affected by spasticity, and (d) mild involvement of diplegia, ataxia/athetosis or hemiplegia, also called minimal impairment criteria to be eligible in para-sport. 

In para-sports, particularly for CP football, classification is a unique and distinctive element with respect to regular sports that allow a more equitable practice [8]. The purpose of the paralympic classification systems is to promote participation in sport by people with disabilities by minimising the impact of impairment(s) on the outcome of competition [9]. Thus, classification systems aim to ensure that athletes who succeed in paralympic sport do so because they have the most favourable anthropometric, physiological and psychological attributes and have enhanced them to best effect through training and diet; athletes should not succeed simply because their impairment is less severe than that of their competitors. For this reason, the development of evidence-based classification systems is present in the agendas of all international federations whose para-sports are, or are intended to be, in the Paralympic Games. These classifications should be determined for each para-sport through multi-disciplinary research [10]. The impact that different eligible impairments, and their severity, have on the skills demanded in each para-sport should be assessed [9], evaluating, in turn, the impact they would have on sports performance [11].

It has been defined that vertical jumping ability is one of the relevant variables for sports classification with respect to football performed by players with CP [12]. However, in this para-sport, only four studies in the literature have assessed athletes with CP performing vertical jumps. First, a study by Cámara et al. [13], with 13 international para-footballers, analysed the parameters that characterise vertical ground reaction forces during the landing phase of a vertical jump (i.e. counter movement jump, CMJ), demonstrating that the impact of the forefoot with the ground was similar between individuals with and without CP. They also demonstrated that elite CP soccer players exhibit higher values of impact for the rear foot with the ground, suggesting a reduced cushioning capacity during the landing phase. However, the authors of that study did not distinguish between the different CP-Football impairment profiles. Second, Yanci et al. [14] explored the relationships between sprinting, agility and vertical/horizontal jumps in footballers. They did not find significant differences between the dominant and non-dominant legs in vertical jump tests, and the type of impairment was also not considered. The same authors [15] examined anaerobic fitness in a population of 12 football players with CP, using vertical jumping and Wingate tests, and found that it was significantly lower compared with footballers without impairment. Third, Reina et al. [12] conducted a study with 132 international para-footballers, including CMJ as a vertical jump and three horizontal jumps (standing broad jump, triple hop and four bounds for distance). This is the only study that has compared the performance between the abovementioned CP football profiles, and those with a mild impairment had significantly higher scores in all jump tests as compared with players in the lower classes (i.e., those with moderate impairments). Although in football most of the jumps are made in order to challenge for the ball, in the literature there are only studies that have assessed jumping in an analytical manner. There are no studies addressing jumping conditions that occur in football games. Given the need to develop specific classification systems of each para-sport, to assess the impact of impairments on the performance of specific actions of the game, it is necessary to assess how the presence of the ball for a header can condition the performance parameters of vertical jumps.

Therefore, the goals of the present study were: (1) to assess how the modification of jumping conditions (with and without a header) might affect the kinematic and kinetic parameters of vertical jump performance; and (2) to determine the strength of the relationships between the functional profiles of the players and their vertical jumping ability, both with and without a header. 

## 2. Materials and Methods

### 2.1. Participants

A group of 13 male football players with CP participated in the present study, representing the different CP football profiles of spastic diplegia (n = 3), ataxia/athetosis (n = 3), spastic hemiplegia (n = 5) and minimum impairment (n = 2) (Table 1), according to the official classification of functional profiles of the International Federation of Cerebral Palsy Football (IFCPF). The players with spastic diplegia and athetosis/ataxia represent the “lower” sport classes for the game [8]. All of the players were competing in the 2015–2016 Spanish national league, and six of them were regularly involved in the national CP football team with a training frequency of three sessions per week. All the players participated voluntarily in the study and had a confirmed sport class. Approval by the Miguel Hernández University (Elche, Spain) review board (Office for Projects Evaluation) was obtained before the study began (Ref. DPS.RRV.01.14). 

### 2.2. Procedures

Data collection was conducted in February 2016, that is, the time of the year when the Spanish CP Football championships took place. Participants were recruited following an invitation to the teams competing in the championships and a total of 19 players indicated that they wished to participate in the study. To have balanced subgroups, a sample of 13 was finally recruited for the study, considering representation of the different CP football profiles, the affected body side and the available time for data collection. All the players performed ten counter movement jumps with arms swing (CMJAS), five without and five with heading a ball. The two types of jumps, with and without the ball action, were executed in a balanced order among the participants. The time between jumps was set to 30 s and there was 2 min between jumping conditions (without and with header). All the players conducted a 6-min warm-up including running, change of direction exercises and stretching. After this, the players received instructions about the protocol, and they were instructed about the mechanics of jumping performance. A force platform was located in a central space with respect to the seven kinematic analysis cameras (T10 series, Vicon MX, Oxford, UK). The platform was connected to the motion capture system to obtain synchronised records. Data collection was conducted in an indoor sports hall located at a Sports Research Centre to guarantee the quality of the data collected by controlling the light conditions and instruments placements. 

### 2.3. Test Battery

**Figure 1:**
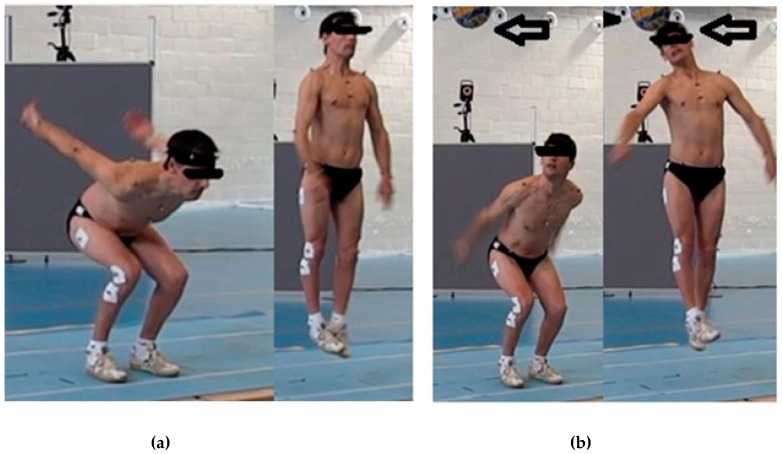
Jumping without (**a**) and with (**b**) a header.

Counter movement jumping with arms swing (CMJAS) and CMJAS with a header. The athletes performed two variants of vertical jump in a balanced way. In the CMJAS, the athletes began the movement from an upright position, then descended and climbed again as quickly as possible. To make the jump as ecological as possible, the swinging of the arms and the choice of the preferred degree of flexion and descent speed were allowed. The CMJAS were then performed with a header. A ball was placed above the head of the participants at a distance of 20% higher than the players’ heights and slightly shifted to the right (Figure 1). The ball was similar to the one used in official games, but softer to prevent problems when heading the ball. The height of 20% above the players’ height was considered adequate after several pilot tests prior the study, to challenge the jump to be a participant’s maximum high and, at the same time, attainable. The athletes had to perform CMJAS with the same characteristics as the previous one, and also hit the ball with the head during the jumps. 

### 2.4. Data Extraction

#### 2.4.1. Kinematic Analyses 

**Figure 2:**
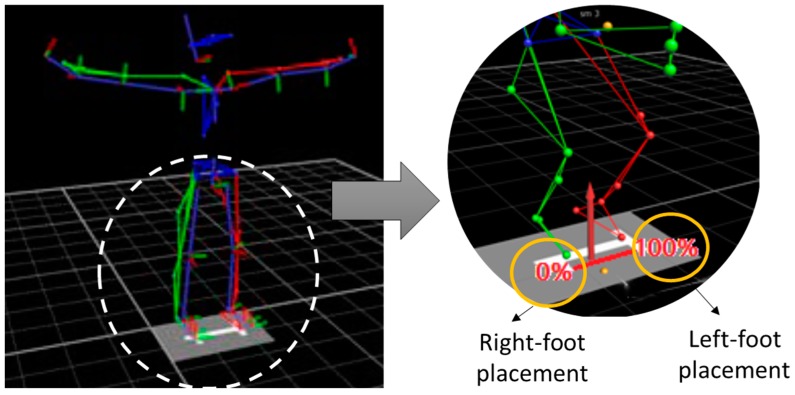
Assessment of postural control of the jumps during the application of force.

Prior to data collection, 35 passive reflective markers (Vicon, Oxford, UK) of 12 mm diameter were placed on each participant, adjusted to the “plug-in-gait full body” model [16]. The same experienced researcher placed the markers to all participants. We used a 3D optical motion capture system consisting of 7 cameras (T10 series, Vicon MX, Oxford, UK) recording at a 200 Hz frequency and synchronized through Nexus v1.7 software (Vicon MX, Oxford, UK). In order to obtain the trajectories of the markers during the jumps, all the records were reviewed, and the gaps filled in for sporadic losses of the markers using the tools of the Nexus software. Subsequently, a double 2nd order Butterworth filter with a cut-off frequency of 6 Hz was applied to the trajectories and the plug-in-gait model was reconstructed to calculate the joint angles from the positions of the markers. The angles of the shoulders, hips, knees, and ankles were calculated in the sagittal plane for both the dominant (D) and the non-dominant (ND) sides. Both D and ND sides were identified according to the following criteria: i) the leg with a higher degree of impairment (e.g., spasticity) and/or ii) the leg used for passing and kicking as per players report. As relevant variables of the angles of the lower limbs, the values were extracted at the moment of maximum knee flexion and at the time of take-off, determined by the decrease in the vertical force below a threshold of 20 N. In addition, we calculated the range of motion (ROM) in the shoulders during the jumps. As a postural control variable in the jumps, we measured the symmetry of the location of the centre of pressures at the instant of maximum vertical force. The width of the support base was defined as a reference, delimited by the toe markers (placed at the 2nd metatarsal bones), assigning 0% to the position of the right foot and 100% to that of the left foot (Figure 2). It was considered that a balanced jump was one in which the centre of pressure was around 50% with respect to the support base. The average of the values of each variable in the five jumps performed with and without a header was used to analyse the data. In addition, the index of asymmetry between the D and the ND sides in each of the variables was calculated using the following formula: Asymmetry = (ND − D) × 100/D [17]. 

#### 2.4.2. Kinetic Analyses 

A Kistler 9286AA triaxial dynamometric platform (Kistler Instruments, Winterthur, Switzerland), measuring 600 × 400 mm, with a sampling frequency of 1000 Hz, was used to record the kinetic data relating to force. The data obtained with the force platform were used to calculate the following variables in the jumps: jump height (Vt^2^/2g: Vt = velocity at take-off instant and g = gravity acceleration (9.8 m/s^2^)); maximal force (maxF: maximum force achieved over the force-time curve during the jump); and rate of force development (RFD: maximum force that occurred over the first derivative of the force-time curve in the concentric phase). The velocity was obtained from the integral force with respect to time using the trapezoidal rule [18]. Five attempts were made for each jump, and all of them were analysed. Their average was calculated as a representative value of the performance. The kinematics and kinetics data collection systems were synchronised through an analogical integrative system. The recording was activated with a trigger plugged to both systems and a LED light. 

### 2.5. Statistical Analysis 

The results are presented as mean ± standard deviation (SD). Prior to statistical analyses, a Kolmogorov-Smirnov normality test was conducted to confirm the pertinence of parametric techniques. A *t*-test of related measurements was used to calculate the differences between the CMJAS without and with a header (i.e., independent variable), both for the kinematic and kinetic variables (i.e., dependent variables). In addition, the effect size was calculated using Cohen’s *d*. We used Hedges correction (*d_g_*) because the sample was small [19]. The interpretation of this effect size was made according to the following values: < 0.25 = trivial; 0.25–0.5 = small; 0.5–0.8 = moderate; > 0.8 = large [20]. In order to measure the degree of relationship between the functional profile of the players (i.e., spastic diplegia, ataxia/athetosis, spastic hemiplegia and minimum impairment) (i.e., nominal variable) and the kinematic and kinetic variables in the two jumps (CMJAS with and without a header) (i.e., continuous variables), we used Spearman’s correlation coefficients (Rho), and 90% confidence limits (90% CL) [21]. The data were analysed using the Statistical Package for Social Science (version 23 for Windows, SPSS Inc, Chicago, IL, USA). The level of significance was set at *p* < 0.05. Despite the fact that in some cases, the differences showed a p value > 0.05, whereas *d_g_* was large (*d_g_* > 0.8), was considered practical differences. 

## 3. Results

Table 2 illustrates the results obtained by all the participants for the kinematic variables and asymmetry (D-ND), both in the CMJAS and in the CMJAS with a header. The angles measured at the moment of maximum hip flexion, both for the D and ND sides, were significantly lower in the CMJAS with a header in comparison to the CMJAS without a header (*d_g_* = 0.75–0.79; *p* < 0.01). With respect to the angle at the moment of maximum flexion of the knee of the D leg (*d_g_* = 1.04; *p* < 0.05) and the ND leg (*d_g_* = 1.15; *p* = 0.08), a lower angle was also observed in the CMJAS with a header in comparison to the CMJAS without a header. However, the angle of the ankle of the D leg measured at the time of take-off was greater in the CMJAS with a header than in the CMJAS without a header (*d_g_* = −0.71; *p* < 0.05). With respect to asymmetry (D-ND), only significant differences were observed between the CMJAS and the CMJAS with a header in the asymmetry of ankle extension (*d_g_* = −1.06; *p* < 0.05).

The results obtained in the kinetic variables for CMJAS and CMJAS with a header are shown in Table 3. The results indicated higher RFD in the CMJAS with a header in comparison to the CMJAS without a header (*d_g_* = −1.24; *p* < 0.05). No significant differences were observed between both jumps in height and maxF (*d_g_* = −0.56 – 0.61; *p* > 0.05).

The functional profile of football players (i.e., diplegia, ataxia/athetosis, hemiplegia, and minimal impairment) correlated significantly with the angle measured at the moment of maximum knee flexion in the CMJAS with a header in the D (Rho = 0.80 ± 0.20 CL, *p* < 0.01) as ND sides (Rho = 0.58 ± 0.34 CL, *p* < 0.05) (Table 4). In addition, a significant correlation was observed between the functional profiles and the angles of flexion of the ankles in the ND sides (Rho = 0.78 ± 0.22 CL; *p* < 0.01). No significant correlation (*p* > 0.05) was observed between the functional profile of the players and the kinetic variables analysed in the two types of jumps (CMJAS with and without a header). 

## 4. Discussion

Vertical jumping has been widely addressed by scientists and coaches to assess the explosive qualities of lower limbs in athletes [14,22,23]. Although the ability to generate explosive force can also be fundamental to assess the performance of football players with CP, given that the power of the lower limbs is decisive in numerous actions of the game [12]. Few studies have analysed this aspect in football players with CP [12,13,14,15]. All the studies published that have assessed vertical jumping ability in football players with CP used tests that are not specific for the actions of the game. Taking into account that, in most cases, vertical jumps are performed to head the ball, further studies on the performance of football players with CP should be conducted assessing vertical jumps more in line with the actual actions of the game. In this sense, the goals of the present study were: on the one hand, to analyse, from a kinematic and kinetic perspective, two jumping conditions (CMJAS and CMJAS with a header) in football players with CP; and, on the other, to analyse whether there was a correlation between the functional profile of the players and vertical jumping ability with and without a ball.

Studies published in the scientific literature addressing vertical jumping ability in football players with CP have assessed vertical jumps from a kinetic perspective [12,13,14,15]. So far, there are no studies addressing football players with CP to assess vertical jumping ability using kinematic methods, as well as more specific vertical jumps performed in the game, such as CMJAS with a header. The results obtained in the present study have indicated differences between CMJAS and CMJAS with a header performed by players with CP. It has been observed that CMJAS with a header implies a lower angle of the hip measured at the moment of maximum flexion in both the dominant and the non-dominant side. In addition, in the jumps with a header, the players exhibited a lower angle of the knee measured at the moment of maximum flexion. However, in the jumps with a header, the players reached higher values of ankle extension. These results indicate that there were differences in the kinematics of jumps between CMJAS and CMJAS with a header. According to the results obtained, players with CP tend to flex less in the eccentric phase of CMJAS with a header in comparison to CMJAS, perhaps as a preparatory mechanism due to the need to direct the body towards the ball. On the other hand, also from the perspective of kinematic analysis, it has been observed that the asymmetries (D-ND) of the ankle flexion are accentuated in jumps with a header in comparison to CMJAS. These results partially coincide with those obtained by Wagner et al. [24]. These authors also observed that the use of the ball, simulating a header, would cause greater asymmetry in the jumps. It seems that football players with CP increase the asymmetry of the ankle flexion in jumps with a header. It would be reasonable to think that the precision demanded by a jump with a header could condition these asymmetries, and the players could make adjustments in the direction of their jumps at the time of the take-off. These differences between both jumps should be taken into account by coaches and physical trainers of football players with CP in order to adapt the training to the specific demands of football. 

From a kinetic perspective, another contribution of the present study is the analysis of the kinetic differences between CMJAS and CMJAS with a header. Although no significant differences were observed between both jumps in the height reached and in the maxF made by the players, the jumps with a header promoted higher RFD values. Taking into account that the RFD is expressed as the relationship between the forces generated in a given time [25,26], the results obtained in the present study seem to indicate that jumps with a header may require greater explosive neuromuscular capacity. Therefore, our results indicate that, from a neuromuscular perspective, jumps with a header can be more demanding (greater RFD) than CMJAS, especially when they have been performed with lower angles of flexion and extension of the hips and knees. In this sense, it may be relevant to know the impact that a large number of jumps with a header to score a goal or challenge for the ball would have, for example, on a game or training of football players with CP with respect to their neuromuscular fatigue. Previous researchers have stated that players with CP have lower capacity to produce force in short periods of time [26,27]. This way, it would be interesting to know whether training vertical jumps with a header—which, as observed in the present study, promoted higher RFD in football players with CP—might promote positive effects in comparison to training jumps without a header.

Regarding the functional profiles of this para-sport, a previous study had observed that there were differences in vertical jumping ability depending on the functional profiles [12]. Specifically, these researchers observed that vertical jumping ability was greater in football players with less limitation in comparison to players with greater limitation. It is worth mentioning that those results are partially in line with the results obtained in the present study. Regarding CMJAS, no significant correlations were observed between the functional profile of the players and the kinetic or kinematic variables. However, when performing CMJAS with a header, the players with less limitation exhibited greater angle of knee flexion, both on the D and ND sides, as well as greater angle of ankle flexion in the ND side. The results obtained in the present study indicate that jumping patterns could be partially affected by functional profiles. The correlations in some of the kinematic variables could indicate the impact of a limitation on vertical jumping ability, especially when the jump requires a fast eccentric/concentric action [28]. This limitation in jumping ability may be related to limitations in other actions of the game, such as sprint or changes of direction [29,30], specific actions, and determinants of performance in football.

However, these results of the present study should be taken with caution, mainly due to the small sample used, given that no significant correlations were observed between the functional profile of the players and the kinetic variables (CMJAS and CMJAS with a header). These results are in line with those obtained by two previous studies, also conducted with small sample sizes (< 15 football players with CP), in which no correlations were observed between the functional profiles and vertical jumping ability [14,15]. Like in other studies [15,31], Spearman’s correlation coefficient analysis has been used to analyze the relationship between the players´ functional profile (i.e. nominal variable) and the kinematic and kinetic parameters of their performance (i.e. continuous variables). Due to this analysis may have some limitations, it is necessary to explore other more robust statistical analyses to determine the abovementioned associations. However, the only study conducted with a large sample of players observed differences in vertical jumping ability between players with different functional profiles [12]. Possibly, the absence of associations between the functional profile of the players and some kinetic and kinematic variables in the present study may have been conditioned by the sample size and by the correlational analysis used. Therefore, further studies are needed to help clarify these contradictory results. In addition, it was not possible to analyse differences in the variables assessed between players with different functional profiles. The results obtained in the present study may have been conditioned by integrating participants with different functional profiles and, therefore, with different neuromuscular and performance characteristics. Further studies should analyse vertical jumping ability with and without a header, both from a kinematic and kinetic perspective, with a greater number of football players with CP. 

## 5. Conclusions

Significant differences were observed in the performance of CMJAS with and without a header, with less hip and knee flexion and greater ankle extension in CMJAS with a header. This way, the combination of these differences in the kinematic (greater flexion in the eccentric phase of the jump), together with the greater RFD found in the concentric phase of the jump, seems to demonstrate greater explosiveness or neuromuscular involvement when performing CMJAS with a header. With respect to the relationship of the functional profiles in this para-sport, the relationships obtained with respect to CMJAS with a header confirm the different demands of vertical jump performance and its possible implications in the training of football players with CP or even in the game. 

## Figures and Tables

**Table 1:** Characteristics of the study participants.

Impairment Profile	N	Age (year)	Body Mass (kg)	Height (cm)	BMI (kg/m^2^)
Diplegia	3 (2L-1R)	27.0 ± 4.4	66.5 ± 11.6	169.6 ± 1.5	23.1 ± 3.3
Ataxia/Athetosis	3 (1L-2R)	21.0 ± 1.7	62.6 ± 4.1	170.6 ± 9.3	21.5 ± 1.1
Hemiplegia	5 (2L-3R)	27.8 ± 4.6	74.6 ± 9.1	178.2 ± 10.1	23.7 ± 3.4
Minimal Impairment	2 (2R)	35.2 ± 1.4	72.2 ± 11.5	176.0 ± 7.1	23.1 ± 1.8
Overall	13 (5L-8R)	27.7 ± 5.7	68.9 ± 5.4	173.6 ± 4.2	22.8 ± 0.9

L = left-handed, R = right-handed, BMI = body mass index.

**Table 2:** Comparisons between CMJ with and without a header, considering the body region and asymmetry.

CMJAS Kinematics		without a Header			with a Header			t	*p*	*d_g_*
		M	±	SD	M	±	SD			
Dominant Side (Ds)	Hip (°) (1)	90.0	±	16.9	76.3	±	17.1	3.30	0.007 **	0.75
	Knee (°) (1)	92.1	±	13.7	76.8	±	21.0	2.85	0.016 *	1.04
	Ankle Flexion (°) (1)	34.9	±	7.7	36.0	±	12.4	−0.33	0.746	−0.13
	Ankle Extension (°) (2)	20.6	±	6.7	25.7	±	9.6	−2.89	0.015 *	−0.71
	Shoulder Extension (°) (1)	−39.8	±	19.4	-32.1	±	19.6	−1.60	0.139	−0.37
	Shoulder Flexion (°) (2)	53.6	±	17.3	54.1	±	19.6	−0.14	0.893	−0.03
	Shoulder ROM (°)	87.1	±	31.1	77.9	±	40.6	0.96	0.357	0.28
Non-Dominant Side (NDs)	Hip (°) (1)	93.0	±	22.9	73.5	±	17.6	3.48	0.005 **	0.79
	Knee (°) (1)	92.9	±	10.2	80.3	±	21.2	1.95	0.076	1.15
	Ankle Flexion (°) (1)	31.9	±	8.3	28.4	±	8.1	1.87	0.089	0.39
	Ankle Extension (°) (2)	18.9	±	6.2	17.7	±	7.6	1.28	0.228	0.18
	Shoulder Extension (°) (1)	−28.4	±	19.8	−26.3	±	20.4	−0.55	0.597	−0.10
	Shoulder Flexion (°) (2)	50.5	±	16.7	49.7	±	15.1	0.16	0.877	0.05
	Shoulder ROM (°)	74.6	±	25.2	68.9	±	38.3	0.76	0.466	0.21
Asymmetry (Ds – NDs)	Hip (°) (1)	−2.9	±	12.8	2.8	±	9.5	−1.34	0.207	−0.41
	Knee (°) (1)	−0.8	±	6.2	−3.5	±	16.7	0.71	0.495	0.41
	Ankle Flexion (°) (1)	3.0	±	8.9	7.6	±	13.8	−1.34	0.208	−0.48
	Ankle Extension (°) (2)	1.6	±	5.6	8.0	±	11.7	−3.07	0.011 *	−1.06
	Shoulder Extension (°) (1)	−11.5	±	15.6	−5.8	±	13.6	−1.34	0.207	−0.34
	Shoulder Flexion (°) (2)	3.1	±	13.9	4.4	±	20.4	−0.24	0.815	−0.09
	Shoulder ROM (°)	12.5	±	27.7	9.0	±	24.2	0.62	0.546	0.12

M = mean; SD = standard deviation; CMJAS = counter movement jump with arms swing; ROM = range of movement; (1) angles measured at the moment of maximum flexion; (2) angles measured at the time of take-off; * *p* < 0.05; ** *p* < 0.01 significant differences between with and without header values; *d_g_* = effect size.

**Table 3:** Comparisons between CMJAS with and without a header for the kinetics variables.

CMJAS Kinetics	without a Header			with a Header			t	*p*		*d_g_*
	M	±	SD	M	±	SD				
Height (cm)	38.4	±	11.5	30.9	±	6.3	2.11	0.058		0.61
maxF (N)	2.4	±	0.5	2.7	±	0.3	−1.62	0.134		−0.56
RFD (N/s)	30.9	±	15.9	52.1	±	47.4	−2.24	0.047	*	−1.24

M: mean; SD: standard deviation; CMJ: counter movement jump; maxF: maximal force, RFD: rate of force development; * *p* < 0.05; ** *p* < 0.01 significant differences between with and without header values.

**Table 4:** Correlation (Spearman’s Rho ± 90% CL) between the functional profile of the players and the kinematic and kinetic variables of the CMJAS and the CMJAS with a header.

CMJ Kinematics		without a Header	with a Header
		Rho Spearman ± 90% CL	Rho Spearman ± 90% CL
Dominant Side (Ds)	Hip (°) (1)	NS	NS
	Knee (°) (1)	NS	0.80 ± 0.20**
	Ankle Flexion (°) (1)	NS	NS
	Ankle Extension (°) (2)	NS	NS
	Shoulder Extension (°) (1)	NS	NS
	Shoulder Flexion (°) (2)	0.79 ± 0.21**	NS
	Shoulder ROM (°)	NS	NS
Non-Dominant Side (NDs)	Hip (°) (1)	NS	NS
	Knee (°) (1)	NS	0.58 ± 0.34*
	Ankle Flexion (°) (1)	NS	0.78 ± 0.22**
	Ankle Extension (°) (2)	NS	NS
	Shoulder Extension (°) (1)	NS	NS
	Shoulder Flexion (°) (2)	NS	NS
	Shoulder ROM (°)	NS	NS
Asymmetry (Ds – NDs)	Hip (°) (1)	NS	NS
	Knee (°) (1)	NS	NS
	Ankle Flexion (°) (1)	NS	NS
	Ankle Extension (°) (2)	NS	NS
	Shoulder Extension (°) (1)	NS	NS
	Shoulder Flexion (°) (2)	NS	NS
	Shoulder ROM (°)	NS	NS
	Height (cm)	NS	NS
	maxF (N)	NS	NS
	RFD (N/s)	NS	NS

CL = confidence limit; CMJ = counter movement jump; ROM = range of movement; (1) angles measured at the moment of maximum flexion; (2) angles measured at the time of take-off; maxF: maximal force; RFD: rate of force development; * *p* < 0.05; ** *p* < 0.01 significant correlations.
